# Supplementary material for: Poverty alleviation for people with severe mental health conditions in South Africa: A qualitative study
Source: PLOS Glob Public Health. 2026 Sep 9;6(9):e0006551. doi: 10.1371/journal.pgph.0006551 (PMC13557333; doi:10.1371/journal.pgph.0006551)
Supplement: Supplementary file 2 [file pgph.0006551.s002.docx]

**TOPIC GUIDE FOR ECONOMIC EMPOWERMENT FOCUS GROUP DISCUSSION**

**Note for interviewers:**

**Instructions for you are numbered and in BOLD.**

Text for you to read to the participant is in normal text.

| Date of interview |  |
| --- | --- |
| Name of interviewer |  |
| Participant ID |  |

**Introduction**

Thank you for taking time and agreeing to this interview. As you know, people living with mental illness and their caregivers need social support and the PRIZE has been facilitating a programme for this support. We would like to make this programme more widely available, and we want it to fit the needs of people who participate and the facilitators of the groups, so we want to hear about your experiences of the programme and what could be changed or improved.

**Interview Section 1 Financial security and financial literacy**

**Start a discussion with the participant using Question 1 below. Give them time to share their thoughts. Then use the probes to get them to talk more about the topic.**

***Question 1: Please tell me about how money and your financial situation affects you and your family?***

Probe: What does financial security mean to you, how would it look and feel to have financial security?

Probe: Can you talk about some of the things that make it hard to have financial security?

Probe: Can you tell us about any experience you’ve had with training on financial literacy - so being given information and skills on how to manage your finances?

Probe: Has anyone ever helped you with managing your finances better and if so can you talk about what they did and how they helped?

Probe: Looking back, what would have been most useful for you or what would you like to know about when it comes to money and finances?

**Interview Section 2 Community experiences of saving and income generating**

**Start a discussion with the participant using Question 2 below. Give them time to share their thoughts. Then use the probes to get them to talk more about the topic.**

***Question 2: Do you know stories or examples of people in your community who have turned things around for themselves financially- could you tell us about these***

Probe: What are the things you think stops people in your community from being able to earn an income?

Probe: Can you tell us about the things you feel stop people from being able to save a part of their income or their grant in your community?

Probe: Can you tell us about any examples in your community of successful group savings collectives or other examples of how people have grouped together to try to make their financial situation better?

Probe: Can you tell us about any efforts in your community where people have grouped together to do income generating activities?

Probe: Can you think of anyone who has used a grant or pension in a creative way to earn extra income – can you tell use about this?

Probe: What types of small income generating projects do you think would be successful in your community – what is needed and what do you think people would buy or pay for?

**Interview Section 3 Acceptability of group savings**

**Explain that a group savings model is where people in a group put a sum of money into a ‘pot’ together each week or month, that is saved and then each member has a chance to use the whole ‘pot’. There are rules as to when and how members can get the money. Start a discussion with the participant using Question 3 below. Give them time to share their thoughts. Then use the probes to get them to talk more about the topic.**

***Question 3:* Can you tell us about what you know or have experienced about group savings schemes or ‘stockvels’?**

Probe: If there was a group saving part of the PRIZE recovery groups, would you feel that you wanted to be part of it. Can you tell us more about why or why not?

Probe: What do you think would be needed for something like this to work in the PRIZE recovery groups?

Probe: Can you talk about some of the things that could be advantages or disadvantages of including this in the PRIZE recovery groups?

**Interview Section 4 Experiences from PRIZE recovery groups**

**Start a discussion with the participant using Question 4 below. Give them time to share their thoughts. Then use the probes to get them to talk more about the topic.**

***Question 4:* The idea of trying to make extra income did come up in some of the groups with the problem solving – can you tell us what happened in your groups?**

Probe: What ideas did people have in your groups for generating an income?

Probe: Did you manage to make any progress on these what were some of the barriers you faced, can you tell us more about this?

Probe: What skills or ideas do you have that you think could be used to try to make an income generating project?

Probe: What would you like to learn about or create?

**Is there anything else you would like to mention that we have not adequately covered in this interview?**

THANK YOU

## Interview guide for recovery group participants on economic empowerment

**Note for interviewers:**

**Instructions for you are numbered and in BOLD.**

Text for you to read to the participant is in normal text.

| Date of interview |  |
| --- | --- |
| Name of interviewer |  |
| Participant ID |  |

**Introduction**

Thank you for taking time and agreeing to this interview. As you know, people living with mental health conditions and their caregivers need social support and the PRIZE study has been facilitating a programme for this support. During the PRIZE programme, many group members talked about how they wished for greater financial freedom and security as part of their recovery. We would like to hear more from you about this to help us improve future recovery groups.

**Interview Section 1 Experiences of financial insecurity**

**Start a discussion with the participant using Question 1 below. Give them time to share their thoughts. Then use the probes to get them to talk more about the topic.**

***Question 1: The PRIZE groups were about recovery for people with mental health conditions and their families, what role do you think finances have in recovery?*** **Amaqela e-PRIZE ebemalunga nokululama kwabantu abaneemeko zempilo yengqondo kunye neentsapho zabo, yintoni indima ocinga ukuba iimali inayo ekululameni?**

Probe: Could you tell me what causes pressure on you and family members when it comes to money? **Ungandixelela ukuba yintoni ebangela uxinzelelo kuwe nakumalungu osapho xa kufikwa kwimali?**

Probe: How do you think this affects you and/or your family members? **Ucinga ukuba oku kukuchaphazela njani wena kunye/okanye amalungu osapho lwakho?**

Probe: How does it affect your planning for the future? **Ikuchaphazela njani ukucwangcisa kwakho ngekamva?**

Probe: if you (**your relative is**) are short of money, how well can (**you**) your family support you (them)?

Probe: how do you cope with experiencing financial strain? **Umelana njani noxinzelelo ngokwezemali?**

Probe: How do you think things could be different in your financial situation and how would this be for your family? **Ucinga ukuba izinto zinokwahluka njani kwimeko yakho yezemali kwaye oku kuya kuba njani kusapho lwakho?**

Ukuba izinto zingohluka kusapho lwakho kwezezimali, zingohluka njani (kuxa kwenzeke ntoni) kwaye oku kungathetha ntoni kuwe nosapho lwakho?

**Interview Section 2 Experiences of disability grants/** **Amava ngezibonelelo zokukhubazeka**

**Start a discussion with the participant using Question 2 below. Give them time to share their thoughts. Then use the probes to get them to talk more about the topic.**

***Question 2: Please tell me about your experiences with disability grants over time./*** **Nceda undixelele ngamava akho ngezibonelelo zokukhubazeka ngokokuhamba kwexesha**

Probe: Can you tell me about how (they) you first got this grant and what happens now (is the process) for you to get this grant? **Ungandixelela malunga nendlela owasifumana ngayo esi sibonelelo kwaye kwenzeka ntoni ngoku ukuze ufumane esi sibonelelo?**

**Probe: Did you have any difficulties with being approved by the SASSA doctor?**

Probe: If there have been times when (they) you didn’t get the grant, can you tell me why you think that happened? What are some of the difficulties you’ve had? **Ukuba kukho amaxesha apho uye awasifumana isibonelelo, ungandixelela ukuba kutheni ucinga ukuba kwenzeke oko? Zeziphi ezinye zeengxaki oye wazifumana?**

Probe: Have you or anyone you know used the disability grant in different ways, for example in a group savings (stokvel) or using it to start a small business? Can you tell us more about this? **Ingaba wena okanye nabani na omaziyo uye wasebenzisa igranti ngeendlela ezahlukeneyo, umzekelo, kwiqela lokulondoloza (stokvel) okanye ukuyisebenzisela ukuqala ishishini elincinci? Ungasixelela ngakumbi malunga noku?**

Probe: To what extent does getting a disability grant affect your motivation to make money or find a job? **Ingaba ukufumana kwakho isibonelelo sokukhubazeka kunegalelo elingakanani ekuzikhuthazeni uzenzele imali okanye ekufumaneni umsebenzi?**

Probe: if you could choose, would you rather be receiving a grant or getting a job? Why?

**Interview Section 3 Experiences relating to income generating activities and savings/ Amava anxulumene nemisebenzi yokwenza ingeniso kunye nokonga.**

**Start a discussion with the participant using Question 3 below. Give them time to share their thoughts. Then use the probes to get them to talk more about the topic.**

***Question 3:* Can you tell me about the kinds of things you have done to try to make your financial situation better?/** ***Ungandixelela ngeentlobo zezinto ozenzileyo ukuzama ukwenza imeko yakho yezemali ibengcono?***

Probe: Was there discussion in your PRIZE groups about anything that could help improve the financial situation of your group members? Can you tell me more about this? **Ingaba bekukho iingxoxo kwiqela lePRIZE malunga nantoni na enokunceda ukuphucula imeko yezemali yamalungu eqela lakho? Ungandixelela ngakumbi ngale nto?**

Probe: Can you tell me about anything you know about groups savings or ‘stokvels’. Have you been part of one, if so, what were the things that worked and what didn’t work so well in your community? **Ingaba ungandixelela ngayo nantoni na oyaziyo ngolondolozo lwamaqela okanye ‘izitokfela’. Ingaba uye waba yinxalenye yaso, ukuba kunjalo, zeziphi izinto ezisebenzayo kwaye yintoni engazange isebenze kakuhle kule ndawo uhlala kuyo?**

Probe: Other than finding work or piece jobs can you tell me about anything you have done to try to make an income. How did this go, what worked and what didn’t work? **Ngaphandle kokufumana umsebenzi okanye amatorho ungandixelela ngayo nantoni na oyenzileyo ukuzama ukwenza ingeniso. Kwahamba njani oku, yintoni eyasebenzayo kwaye yintoni engazange isebenze?**

Probe: Now that you have been in a recovery group for a while, can you tell me about what kinds of things you think would be feasible for you and your group to do to work together to try and make extra income? **Ngoku ubukwiqela lokululama ixesha elide, ungandixelela malunga neentlobo zezinto ocinga ukuba zinokwenzeka kuwe kunye neqela lakho ukuze nisebenze kunye ukuzama ukwenza ingeniso eyongezelelweyo?**

**Interview Section 4 Acceptability of a group savings model**

**Start a discussion with the participant using Question 4 below. Give them time to share their thoughts. Then use the probes to get them to talk more about the topic. Explain that a group savings model is where people in a group put a sum of money into a ‘pot’ together each week or month, that is saved and then each member has a chance to use the whole ‘pot’. There are rules as to when and how members can get the money.**

***Question 4:* Please tell me what you think about adding a group savings part to the PRIZE recovery groups. What is your first impression on this? (What do you think of this?/** ***Nceda undixelele ukuba ucinga ntoni malunga nokongeza isihloko ngamaqela alondolozayo kumaqela okululama e-PRIZE. Uthini umbono ofika kuqala kuwe ngalento? (Ucinga ntoni ngale nto?)***

Probe: What do you think would be the good things about this. What do you think would be the challenging things about this? **Ucinga ukuba zithini izinto ezintle malunga noku. Ucinga ukuba ziziphi izinto ezinokuba ngumcelingeni kule nto?**

Probe: If this group savings was part of the PRIZE recovery groups would you join up – and can you tell me why or why not? **Ukuba eli qela lokulondoloza beliyinxalenye yamaqela okululama e-PRIZE ungajoyina- kwaye ungandixelela ukuba kutheni okanye kutheni kungenjalo?**

Probe: From what you know about other group members do you think others in the group would join up. Why or why not? **Ngokukwaziyo ngamanye amalungu eqela, ucinga ukuba abanye kwiqela bangajoyina? Kutheni okanye kutheni kungenjalo?**

Probe: What else could you imagine doing as part of your PRIZE group to help with your financial situation? **Yintoni enye onokuzibona uyenza njengenxalenye yeqela lakho le PRIZE ukunceda kwimeko yakho yezemali?**

**Is there anything else you would like to mention that we have not adequately covered in this interview?**

THANK YOU
