# Supplementary material for: Poverty alleviation for people with severe mental health conditions in South Africa: A qualitative study
Source: PLOS Glob Public Health. 2026 Sep 9;6(9):e0006551. doi: 10.1371/journal.pgph.0006551 (PMC13557333; doi:10.1371/journal.pgph.0006551)
Supplement: Supplementary file 4 [file pgph.0006551.s004.docx]

**Response to reviewers comments**

We are grateful for the reviewer’s comments. Please find our amendments below noting line numbers refer to the tracked changes version.

**Editor:**

1. As the authors suggest in the introduction and discussion, there are historical and political factors playing a substantial role in the socioeconomic status of certain regions/communities in the country. It could pay off to begin the discussion with this acknowledgment (rather than to close with it before moving to the implications). This would help set the scene that other interventions (perhaps a multi-sectoral/ health in all policies approach) could further improve the potential outcomes.

**Response**: We have moved this text to the beginning of the discussion, ‘Our findings were underpinned by broader socio-political determinants (Burgess et al., 2025). The legacy of apartheid manifested in the spatial segregation of townships from employment opportunities and transport links (Luke 2025), as well as in direct experiences of racial discrimination. Despite strenuous legislative and policy efforts to eliminate racial inequalities in employment and economic status (Busse 2025), substantial inequities still exist in South Africa.” (lines 490-497)

2. it could be helpful to bring some further focus to the objectives of the study, as they are presented in Lines 80-85. It might be too ambitious to aim to bring an in-depth exploration of the experience of poverty.

**Response**: We have reframed the study objectives as follows: “This study aimed to interrogate potential poverty alleviation components for incorporation into future psychosocial interventions for people with SMHC and their caregivers in Eastern Cape, South Africa, including stokvels as a contextually appropriate model. We also sought to understand current engagement with poverty alleviation interventions, including disability grants” (lines 78-85)

3. I would encourage the authors to avoid presenting points raised by the participants as more than that. For example, in line 203, the authors explain that "poverty led to alcohol and substance use, which in turn worsened family and social relationships." It would help to use wording that shows this was the perception of the participants, rather than an undisputed fact. Same, for example, when explaining that absence of financial support was due to "high levels of poverty amongst all in these neighbourhoods, mental illness stigma or a general lack of solidarity."

**Response**: We have amended the text to, “Some participants perceived that poverty led to alcohol and substance use” (lines 218-19) and “Participants reported that this was due to high levels of poverty amongst all in these neighbourhoods, mental illness stigma or a general lack of solidarity” (lines 270-71).

**Reviewer #1:** 
General Comments
The manuscript is well written and presents a coherent narrative linking poverty, social exclusion, disability grants, savings groups, and income-generation opportunities among people with SMHC and caregivers. The thematic structure is generally clear. However, the paper occasionally moves beyond the qualitative data to make strong policy recommendations that are insufficiently supported by the study findings. Greater methodological transparency and analytical depth are needed.

**Response**: Thank you for this feedback.

1. Title of the study
   The title "A qualitative study exploring opportunities for poverty alleviation interventions amongst people with severe mental health conditions in Eastern Cape, South Africa" is unnecessarily long and contains redundant phrases such as "A qualitative study exploring opportunities". Here are some shorter alternatives that retain the core meaning: I therefore recommend: Poverty Alleviation for People with Severe Mental Health Conditions in South Africa: A Qualitative Study

**Response**: we have amended to the suggested title.

2. Abstract
   Lines 13–40
   the author was not specific of the study design used, however, it is very important to categorically state that. The results section of the abstract is overly detailed for a qualitative study. The extensive description of all four themes could be condensed to improve readability.

**Response**:

We have amended to, “We conducted a qualitative study involving two focus group discussions and 12 in-depth interviews”

We have reduced the length of the results in the abstract.

3. Study rationale and objectives
   Lines 80–85
   The study objective is broadly stated. The authors should provide clearer research questions and explicitly explain how examining poverty experiences differs from assessing the acceptability of poverty-alleviation interventions. These appear to be two distinct objectives that require clearer alignment throughout the manuscript.

**Response**: We have reframed the study objectives as follows: “This study aimed to interrogate potential poverty alleviation components for incorporation into future psychosocial interventions for people with SMHC and their caregivers in Eastern Cape, South Africa, including stokvels as a contextually appropriate model. We also sought to understand current engagement with poverty alleviation interventions, including disability grants” (lines 78-85)

4. Sampling and representativeness
   Lines 108–119
   The rationale for selecting only participants from the PRIZE trial should be strengthened. Participants who had previously engaged with recovery groups may have different perspectives from people with SMHC in the wider community. This potential selection bias should be discussed more explicitly as a limitation.

**Response**: Although all particpants were PRIZE trial participants, we included both those who had participated in recovery groups and those who were in the control arm. We have added the following text to clarify this:

“The rationale for including participants from the PRIZE feasibility trial was that we felt recovery group participants would be well placed to share insights on how poverty alleviation interventions could be incorporated into psychosocial interventions in practice, which was the primary study objective. However we also included PRIZE control arm participants (i.e. who had not participated in recovery groups) to gain a broader perspective on what would work.” (lines 113-118)

“Six (43%) participants living with SMHC and seven (54%) caregivers had participated in recovery groups as part of the PRIZE trial.” (lines 176-177)

We have added a row indicating trial arm to Table 2 (participant characteristics).

5. Data collection and saturation
   Lines 121–141
   The authors state that saturation was achieved when consecutive interviews yielded no new thematic content; however, no evidence is provided regarding how saturation was assessed or documented. More detail is required to enhance methodological rigour.

**Response**:

We have added the following sentence, “BR, CBS and LA discussed emerging thematic content at intervals of 2-3 interviews.” (lines 146-148)

6. Reflexivity and researcher influence
   Lines 133–140 and 144–156
   The interviewer had previous contact with participants through the PRIZE process evaluation. The manuscript does not adequately discuss how this prior relationship may have influenced data collection, participant responses, or interpretation. A stronger reflexivity statement is needed.

**Response**: We have added the following reflexivity statement:

“The interviewer (BR) had previous contact with recovery group participants (n=13) through observations made as part of the PRIZE process evaluation. It is possible that this familiarity with the interviewer may have meant participants were more willing to share honest accounts of difficult circumstances. However they may also have felt more obliged to be optimistic about potential future amendments to recovery groups. As an isiXhosa-speaking researcher familiar with the study context, BR was able to build rapport and facilitate interviews in participants' preferred language. She remained mindful that prior contact could influence participants' responses and therefore adopted a neutral, non-judgemental interviewing approach, using open-ended questions and reflexive practice to minimise the influence of her assumptions and encourage participants to share both positive and negative experiences. BR did not have prior contact with the control arm participants (n=14). In analysing the data, LA brought the perspective of a white, British, public health doctor and researcher who has previous experience living and working in Ethiopia. CBS is white South African researcher, with institutional affiliation and academic training, as well as experience of living and working in other African countries and of conducting research in a variety of South African settings. These perspectives were complemented by those of a diverse team of co-authors.” (lines 163-172)

7. Data collection reporting
   Lines 138–139
   The sentence reporting interview duration appears incomplete and should be corrected for clarity.

**Response**: This has been corrected as follows:

“Caregiver IDIs lasted between 26 and 44 minutes, people living with SMHC IDIs lasted between 12 and 53 minutes and FGDs lasted 76 and 97 minutes.” (lines 142-144)

8. Participant characteristics
   Lines 160–167
   The extreme gender imbalance should be acknowledged more explicitly. Nearly all participants with SMHC were male, whereas all caregivers were female, which may have shaped the findings.

**Response**: we have added the following text to the limitations “There was substantial gender imbalance in both people with SMHC- all but one of whom was male- and caregivers- all of whom were female. This may have skewed our findings and mean they have limited transferability to women with SMHC, given the important influence of gender in mental health recovery (Asher et al., 2026)” (lines 696-699)

9. Analytical depth
   Lines 172–466

The findings are rich but largely descriptive. Several themes, particularly those concerning crime, stigma, social exclusion, and community fragmentation, would benefit from deeper interpretation beyond participants’ accounts. The analysis could engage more critically with structural determinants of poverty and mental illness rather than primarily describing experiences.

**Response**: Our aim was not to produce a critical structural analysis of poverty, crime, or exclusion, but to generate an inductive thematic account grounded in participants’ experiences. While we applied a socio‑political determinants lens, the analysis remained experience‑led rather than theoretically driven. We have however added some additional points as follows

“These accounts illustrate how individual experiences of financial insecurity are shaped by broader structural conditions- such as racialised labour markets, community violence, and entrenched poverty- which limit opportunities and reinforce cycles of exclusion.” (lines 295-297)

And “Taken together, these mixed experiences show how group savings are influenced not only by individual behaviour but by wider structural conditions such as community violence, poverty, mistrust, and social fragmentation, which influence whether stokvels feel viable or safe.” (lines 420-423)

10. Policy recommendations exceed data
    Lines 519–646
    The four proposed intervention models are interesting but appear partly speculative. The qualitative data support participants’ views on savings groups and income generation, but the discussion occasionally presents implementation pathways, scalability, and sustainability considerations that were not directly explored in the interviews. The authors should distinguish more clearly between findings and authors’ interpretations.

**Response**: We have amended the text as follows, ‘Based on our results and our knowledge of the wider literature, we have identified four potential approaches to providing a poverty alleviation component as an adjunct to psychiatric care and psychosocial interventions, such as PRIZE recovery groups, that would be suitable for low-resource settings. Drawing on both our data and our wider experience, we speculate that each approach differs with respect to (i) potential impact on financial insecurity and/or mental health, (ii) scalability and sustainability, and (iii) safety and ethical concerns (see figure 1).” (lines 544-548)

11. Limitations
    Lines 665–675
    The limitations section should additionally acknowledge limited transferability beyond the township settings studied and the predominance of Black African participants.

**Response**: We have added the following text to the limitations, “Finally we note there may be limited applicability of our findings to settings other than township contexts”. (lines 700-701). We do feel our results are relevant beyond Black African populations to other populations living in very low resource contexts.

**Reviewer #2:** This is a well-conceived and carefully executed qualitative study that makes a meaningful contribution to the field. The bidirectional framing of the poverty-mental health relationship is appropriately grounded in the literature, and the four approaches typology presented in the Discussion and Figure 1 is a genuine conceptual contribution that will be useful to researchers, and other professionals working in similar low-resource contexts.

**Response**: Thank you for this positive feedback

Nevertheless, a few issues require attention:
1. the lines 138-139 have an incomplete sentence (between...?).

**Response**: This has been amended to “Caregiver IDIs lasted between 26 and 44 minutes, people living with SMHC IDIs lasted between 12 and 53 minutes and FGDs lasted 76 and 97 minutes.” (lines 142-144)

2. the operationalisation of data saturation is stated but not described.

**Response**: We have added the following, “BR, CBS and LA discussed emerging thematic content at intervals of 2-3 interviews. Data saturation was considered reached when consecutive interviews yielded no novel thematic content.” (lines 146-147)

3. the interview and focus group topic guide should be uploaded as a Supporting information file, both for methodological transparency and to complete COREQ.

**Response**: These have been added as Supporting Information

4. Additionally, theme 3 is titled "conditional hope" in the abstract but "tentative hope" in the results - please make this consistent.

**Response**: This has been corrected to “tentative hope” throughout.

5. The authors may also add a brief reflexivity statement addressing the interviewer´s positionality.

**Response**: We have added the following reflexivity statement:

“The interviewer (BR) had previous contact with recovery group participants (n=13) through observations made as part of the PRIZE process evaluation. It is possible that this familiarity with the interviewer may have meant participants were more willing to share honest accounts of difficult circumstances. However they may also have felt more obliged to be optimistic about potential future amendments to recovery groups. As an isiXhosa-speaking researcher familiar with the study context, BR was able to build rapport and facilitate interviews in participants' preferred language. She remained mindful that prior contact could influence participants' responses and therefore adopted a neutral, non-judgemental interviewing approach, using open-ended questions and reflexive practice to minimise the influence of her assumptions and encourage participants to share both positive and negative experiences. BR did not have prior contact with the control arm participants (n=14). In analysing the data, LA brought the perspective of a white, British, public health doctor and researcher who has previous experience living and working in Ethiopia. CBS is white South African researcher, with institutional affiliation and academic training, as well as experience of living and working in other African countries and of conducting research in a variety of South African settings. These perspectives were complemented by those of a diverse team of co-authors.” (lines 164-172)

6. Also, to acknowlegde in the Limitations that the near-exclusive recruitment of male participants SMHC may limit the transferability of findings to women living mental illness.

**Response**: We have added the following text “There was substantial gender imbalance in both people with SMHC- all but one of whom was male- and caregivers- all of whom were female. This may have skewed our findings and mean they have limited transferability to women with SMHC, given the important influence of gender in mental health recovery (Asher et al., 2026).” (lines 696-700)

7. The description of the participants, after quotes, could be uniformized.

**Response**: This has been corrected.

8. Theme 1b describes that receiving a disability grant causes intra-family conflict, yet when discussing option 3, the authors suggest that family may be the most appropriate unit of intervention. Isn´t this a contradiction? Perhaps a more comprehensive description of family involvement throughout the paper.

**Response**: We have added the following explanatory points (underlined), “As the financial security of people living with SMHC and caregivers is so highly linked, and in low-resource contexts it is common to live in the same household, we suggest that the family may be the most appropriate unit of intervention delivery (Dijkstra et al., 2025). However this would need to be done in a way that minimised potential family conflict, such as that identified through the receipt of disability grants in our study.” (lines 618-620)

9. line 393 - target?

**Response**: This has been corrected.
