# Supplementary material for: Poverty alleviation for people with severe mental health conditions in South Africa: A qualitative study
Source: PLOS Glob Public Health. 2026 Sep 9;6(9):e0006551. doi: 10.1371/journal.pgph.0006551 (PMC13557333; doi:10.1371/journal.pgph.0006551)
Supplement: Supplementary file 5 [file pgph.0006551.s005.docx]

**Response to reviewers comments**

We are very grateful for the editor’s helpful comments. Please find our amendments below noting line numbers refer to the tracked changes version.

1. The sentence (abstract, theme 1): “Financial insecurity was entrenched due to fractured and violent communities”. This sentence suggests that the community itself is the cause of its financial insecurity. Also, broad descriptors such as ‘violent communities’ have often helped reproduce stigma and exclusion, as they suggest that the whole community is dysfunctional or dangerous. I invite the authors to consider alternative phrasings, e.g.,
   “Financial insecurity was compounded by (violence in the neighbourhoods OR by high rates of violence, which are terms used in the text) and fractured communities. The same comment applies to the same phrasing in the results section, lines 197-198.

**Response**:

We have amended to, “Financial insecurity was entrenched due to violence in the neighbourhoods and fractured communities” (abstract) and “Financial insecurity was entrenched due to violence in the neighbourhoods, fractured communities, cycles of debt and stigma amongst employers.” (lines 192-194).

2. Please also rephrase: “The shadow of crime and violence seemed to seep into multiple aspects of why people become and stay poor.” Consider options such as: Exposure to crime and violence reinforced OR contributed to OR compounded financial insecurity in several ways, such as…”

**Response**: We have amended to “Exposure to crime and violence compounded financial insecurity in several ways” (lines 505-507).

3. Also, the phrase “We found that financial security was crucial to recovery” may need additional context. Perhaps reframing it as participants’ perceptions? For example, we found that participants perceived financial security as crucial to recovery. Similarly, in line 196, the authors say: “Financial security was crucial to recovery”. Could this be equally rephrased as: “Participants perceived financial security as crucial to recovery.”

**Response**: We have amended to, “Participants perceived financial security as crucial to recovery” (abstract) and “Participants perceived financial security as crucial to recovery, yet often experienced substantial financial insecurity, which impacted on wellbeing” (lines 191-193)

4. Consider omitting the term "neutral" when describing the interviewer’s approach in the positionality statement.

**Response**: We have omitted the term neutral (line 165).

5. Discussion’s first sentence (lines 500-501): “This study has presented in-depth findings on the experiences of poverty affecting people with SMHC and their families in South Africa.” As revised at the end of the introduction (lines 80-84), could the authors also revise this first sentence to more accurately reflect the purpose of the study? The claim that the study provides in-depth insight into SMHC’s experiences of poverty does not align with its focus.

**Response**: We have amended to, “This study has presented in-depth findings on potential approaches to poverty alleviation for people with SMHC and their families in Eastern Cape, South Africa” (lines 490-492).
