# Supplementary material for: Poverty alleviation for people with severe mental health conditions in South Africa: A qualitative study
Source: PLOS Glob Public Health. 2026 Sep 9;6(9):e0006551. doi: 10.1371/journal.pgph.0006551 (PMC13557333; doi:10.1371/journal.pgph.0006551)
Supplement: Peer review history

# Peer review: Decision Letter 0

**DOI:** 10.1371/journal.pgph.0006551.r001

**Authors:** Rodrigo Ramalho

7 Jul 2026

PGPH-D-26-01763

A qualitative study exploring opportunities for poverty alleviation interventions amongst people with severe mental health conditions in Eastern Cape, South Africa

PLOS Global Public Health

Dear Dr. Asher,

Thank you for submitting your manuscript to PLOS Global Public Health. After careful consideration, we feel that it has merit but does not fully meet PLOS Global Public Health’s publication criteria as it currently stands. Therefore, we invite you to submit a revised version of the manuscript that addresses the points raised during the review process.

Please submit your revised manuscript by the 6th of August, 2026. If you will need more time than this to complete your revisions, please reply to this message or contact the journal office at globalpubhealth@plos.org. When you're ready to submit your revision, log on to https://www.editorialmanager.com/pgph/ and select the 'Submissions Needing Revision' folder to locate your manuscript file.

Please include the following items when submitting your revised manuscript:

- A letter that responds to each point raised by the editor and reviewer(s). You should upload this letter as a separate file labeled 'Response to Reviewers'.
- A marked-up copy of your manuscript that highlights changes made to the original version. You should upload this as a separate file labeled 'Revised Manuscript with Track Changes'.
- An unmarked version of your revised paper without tracked changes. You should upload this as a separate file labeled 'Manuscript'.

Guidelines for resubmitting your figure files are available below the reviewer comments at the end of this letter.

As the corresponding author, your ORCID iD is verified in the submission system and will appear in the published article. PLOS supports the use of ORCID, and we encourage all coauthors to register for an ORCID iD and use it as well. Please encourage your coauthors to verify their ORCID iD within the submission system before final acceptance, as unverified ORCID iDs will not appear in the published article. *Only*  the individual author can complete the verification step; PLOS staff *cannot*  verify ORCID iDs on behalf of authors.

We look forward to receiving your revised manuscript.

Kind regards,

Rodrigo Ramalho

Academic Editor

PLOS Global Public Health

Journal Requirements:

1. Please include a complete copy of PLOS’ questionnaire on inclusivity in global research in your revised manuscript. Our policy for research in this area aims to improve transparency in the reporting of research performed outside of researchers’ own country or community. The policy applies to researchers who have travelled to a different country to conduct research, research with Indigenous populations or their lands, and research on cultural artefacts. The questionnaire can also be requested at the journal’s discretion for any other submissions, even if these conditions are not met. Please find more information on the policy and a link to download a blank copy of the questionnaire here: https://journals.plos.org/globalpublichealth/s/best-practices-in-research-reporting. Please upload a completed version of your questionnaire as Supporting Information when you resubmit your manuscript.

2. Please provide a detailed online Financial Disclosure statement. This is published with the article. It must therefore be completed in full sentences and contain the exact wording you wish to be published.

a) State the initials, alongside each funding source, of each author to receive each grant. For example: “This work was supported by the National Institutes of Health (####### to AM; ###### to CJ) and the National Science Foundation (###### to AM).”

b) State what role the funders took in the study. If the funders had no role in your study, please state: “The funders had no role in study design, data collection and analysis, decision to publish, or preparation of the manuscript.”

For more information, please go to our submission guidelines:

https://journals.plos.org/climate/s/submission-guidelines#loc-financial-disclosure-statement

3. Please ensure that the funders and grant numbers match between the Financial Disclosure field and the Funding Information tab in your submission form. Note that the funders must be provided in the same order in both places as well.

4. Please update your online Competing Interests statement. If you have no competing interests to declare, please state: "The authors have declared that no competing interests exist."

5. Please include a separate legend or caption for Figure 1 in your manuscript.

6. We have noticed that you have uploaded Supporting Information files, but you have not included a list of legends. Please add a full list of legends for your Supporting Information files before or after the references list.

7. If the reviewer comments include a recommendation to cite specific previously published works, please review and evaluate these publications to determine whether they are relevant and should be cited. There is no requirement to cite these works unless the editor has indicated otherwise.

8. Please review your reference list to ensure that it is complete and correct. If you have cited papers that have been retracted, please include the rationale for doing so in the manuscript text, or remove these references and replace them with relevant current references. Any changes to the reference list should be mentioned in the rebuttal letter that accompanies your revised manuscript. If you need to cite a retracted article, indicate the article’s retracted status in the References list and also include a citation and full reference for the retraction notice.

Additional Editor Comments:

As the authors suggest in the introduction and discussion, there are historical and political factors playing a substantial role in the socioeconomic status of certain regions/communities in the country. It could pay off to begin the discussion with this acknowledgment (rather than to close with it before moving to the implications). This would help set the scene that other interventions (perhaps a multi-sectoral/ health in all policies approach) could further improve the potential outcomes. With this in mind, as mentioned by Reviewer 2, it could be helpful to bring some further focus to the objectives of the study, as they are presented in Lines 80-85. It might be too ambitious to aim to bring an in-depth exploration of the experience of poverty.

One final suggestion for the results section. I would encourage the authors to avoid presenting points raised by the participants as more than that. For example, in line 203, the authors explain that "poverty led to alcohol and substance use, which in turn worsened family and social relationships." It would help to use wording that shows this was the perception of the participants, rather than an undisputed fact. Same, for example, when explaining that absence of financial support was due to "high levels of poverty amongst all in these neighbourhoods, mental illness stigma or a general lack of solidarity."

[Note: HTML markup is below. Please do not edit.]

Reviewers' comments:

Reviewer's Responses to Questions

**Comments to the Author**

1. Does this manuscript meet PLOS Global Public Health’s publication criteria?

Reviewer #1: Partly

Reviewer #2: Partly

**********

2. Has the statistical analysis been performed appropriately and rigorously?

Reviewer #1: Yes

Reviewer #2: N/A

**********

3. Have the authors made all data underlying the findings in their manuscript fully available (please refer to the Data Availability Statement at the start of the manuscript PDF file)?

The PLOS Data policy

Reviewer #1: No

Reviewer #2: Yes

**********

4. Is the manuscript presented in an intelligible fashion and written in standard English?

Reviewer #1: No

Reviewer #2: Yes

**********

Reviewer #1: Review comments

Title: A qualitative study exploring opportunities for poverty alleviation interventions amongst people with severe mental health conditions in Eastern Cape, South Africa

General Comments

The manuscript is well written and presents a coherent narrative linking poverty, social exclusion, disability grants, savings groups, and income-generation opportunities among people with SMHC and caregivers. The thematic structure is generally clear. However, the paper occasionally moves beyond the qualitative data to make strong policy recommendations that are insufficiently supported by the study findings. Greater methodological transparency and analytical depth are needed.

Comments

1. Title of the study

The title "A qualitative study exploring opportunities for poverty alleviation interventions amongst people with severe mental health conditions in Eastern Cape, South Africa" is unnecessarily long and contains redundant phrases such as "A qualitative study exploring opportunities". Here are some shorter alternatives that retain the core meaning: I therefore recommend: Poverty Alleviation for People with Severe Mental Health Conditions in South Africa: A Qualitative Study

2. Abstract

Lines 13–40

the author was not specific of the study design used, however, it is very important to categorically state that. The results section of the abstract is overly detailed for a qualitative study. The extensive description of all four themes could be condensed to improve readability.

3. Study rationale and objectives

Lines 80–85

The study objective is broadly stated. The authors should provide clearer research questions and explicitly explain how examining poverty experiences differs from assessing the acceptability of poverty-alleviation interventions. These appear to be two distinct objectives that require clearer alignment throughout the manuscript.

4. Sampling and representativeness

Lines 108–119

The rationale for selecting only participants from the PRIZE trial should be strengthened. Participants who had previously engaged with recovery groups may have different perspectives from people with SMHC in the wider community. This potential selection bias should be discussed more explicitly as a limitation.

5. Data collection and saturation

Lines 121–141

The authors state that saturation was achieved when consecutive interviews yielded no new thematic content; however, no evidence is provided regarding how saturation was assessed or documented. More detail is required to enhance methodological rigour.

6. Reflexivity and researcher influence

Lines 133–140 and 144–156

The interviewer had previous contact with participants through the PRIZE process evaluation. The manuscript does not adequately discuss how this prior relationship may have influenced data collection, participant responses, or interpretation. A stronger reflexivity statement is needed.

7. Data collection reporting

Lines 138–139

The sentence reporting interview duration appears incomplete and should be corrected for clarity.

8. Participant characteristics

Lines 160–167

The extreme gender imbalance should be acknowledged more explicitly. Nearly all participants with SMHC were male, whereas all caregivers were female, which may have shaped the findings.

9. Analytical depth

Lines 172–466

The findings are rich but largely descriptive. Several themes, particularly those concerning crime, stigma, social exclusion, and community fragmentation, would benefit from deeper interpretation beyond participants’ accounts. The analysis could engage more critically with structural determinants of poverty and mental illness rather than primarily describing experiences.

10. Policy recommendations exceed data

Lines 519–646

The four proposed intervention models are interesting but appear partly speculative. The qualitative data support participants’ views on savings groups and income generation, but the discussion occasionally presents implementation pathways, scalability, and sustainability considerations that were not directly explored in the interviews. The authors should distinguish more clearly between findings and authors’ interpretations.

11. Limitations

Lines 665–675

The limitations section should additionally acknowledge limited transferability beyond the township settings studied and the predominance of Black African participants.

Reviewer #2: This is a well-conceived and carefully executed qualitative study that makes a meaningful contribution to the field. The bidirectional framing of the poverty-mental health relationship is appropriately grounded in the literature, and the four approaches typology presented in the Discussion and Figure 1 is a genuine conceptual contribution that will be useful to researchers, and other professionals working in similar low-resource contexts.

Nevertheless, a few issues require attention:

1. the lines 138-139 have an incomplete sentence (between...?).

2. the operationalisation of data saturation is stated but not described.

3. the interview and focus group topic guide should be uploaded as a Supporting information file, both for methodological transparency and to complete COREQ.

4. Additionally, theme 3 is titled "conditional hope" in the abstract but "tentative hope" in the results - please make this consistent.

5. The authors may also add a brief reflexivity statement addressing the interviewer´s positionality.

6. Also, to acknowlegde in the Limitations that the near-exclusive recruitment of male participants SMHC may limit the transferability of findings to women living mental illness.

7. The description of the participants, after quotes, could be uniformized.

8. Theme 1b describes that receiving a disability grant causes intra-family conflict, yet when discussing option 3, the authors suggest that family may be the most appropriate unit of intervention. Isn´t this a contradiction? Perhaps a more comprehensive description of family involvement throughout the paper.

9. line 393 - target?

**********

what does this mean?). If published, this will include your full peer review and any attached files.

**Do you want your identity to be public for this peer review?**  If you choose “no”, your identity will remain anonymous but your review may still be made public.

For information about this choice, including consent withdrawal, please see our Privacy Policy

Reviewer #1: **Yes:**  Prince Owusu Adoma

Reviewer #2: No

**********

[NOTE: If reviewer comments were submitted as an attachment file, they will be attached to this email and accessible via the submission site. Please log into your account, locate the manuscript record, and check for the action link "View Attachments". If this link does not appear, there are no attachment files.]

# Author comment: Author response to Decision Letter 1

**DOI:** 10.1371/journal.pgph.0006551.r002

10 Jul 2026

Attachment Submitted filename: Response to reviewers comments.docx

# Editor report: Decision Letter 1

**DOI:** 10.1371/journal.pgph.0006551.r003

**Authors:** Rodrigo Ramalho

3 Aug 2026

PGPH-D-26-01763R1

Poverty Alleviation for People with Severe Mental Health Conditions in South Africa: A Qualitative Study

PLOS Global Public Health

Dear Dr. Asher,

Thank you for submitting your manuscript to PLOS Global Public Health.

The resubmitted manuscript has appropriately addressed practically all comments. There are some minor additional suggestions that I hope will not take the authors long to consider and incorporate.

- The sentence (abstract, theme 1): “Financial insecurity was entrenched due to fractured and violent communities”. This sentence suggests that the community itself is the cause of its financial insecurity. Also, broad descriptors such as ‘violent communities’ have often helped reproduce stigma and exclusion, as they suggest that the whole community is dysfunctional or dangerous. I invite the authors to consider alternative phrasings, e.g.,

“Financial insecurity was compounded by (violence in the neighbourhoods OR by high rates of violence, which are terms used in the text) and fractured communities. The same comment applies to the same phrasing in the results section, lines 197-198.

- Please also rephrase: “The shadow of crime and violence seemed to seep into multiple aspects of why people become and stay poor.” Consider options such as: Exposure to crime and violence reinforced OR contributed to OR compounded financial insecurity in several ways, such as…”

- Also, the phrase “We found that financial security was crucial to recovery” may need additional context. Perhaps reframing it as participants’ perceptions? For example, we found that participants perceived financial security as crucial to recovery. Similarly, in line 196, the authors say: “Financial security was crucial to recovery”. Could this be equally rephrased as: “Participants perceived financial security as crucial to recovery.”

- Consider omitting the term "neutral" when describing the interviewer’s approach in the positionality statement.

- Discussion’s first sentence (lines 500-501): “This study has presented in-depth findings on the experiences of poverty affecting people with SMHC and their families in South Africa.” As revised at the end of the introduction (lines 80-84), could the authors also revise this first sentence to more accurately reflect the purpose of the study? The claim that the study provides in-depth insight into SMHC’s experiences of poverty does not align with its focus.

Please submit your revised manuscript by 17 August 2026. If you will need more time than this to complete your revisions, please reply to this message or contact the journal office at globalpubhealth@plos.org. When you're ready to submit your revision, log on to https://www.editorialmanager.com/pgph/ and select the 'Submissions Needing Revision' folder to locate your manuscript file.

Please include the following items when submitting your revised manuscript:

- A letter that responds to each point raised by the editor. You should upload this letter as a separate file labeled 'Response to Reviewers'.
- A marked-up copy of your manuscript that highlights changes made to the original version. You should upload this as a separate file labeled 'Revised Manuscript with Track Changes'.
- An unmarked version of your revised paper without tracked changes. You should upload this as a separate file labeled 'Manuscript'.

Guidelines for resubmitting your figure files are available below the reviewer comments at the end of this letter.

As the corresponding author, your ORCID iD is verified in the submission system and will appear in the published article. PLOS supports the use of ORCID, and we encourage all coauthors to register for an ORCID iD and use it as well. Please encourage your coauthors to verify their ORCID iD within the submission system before final acceptance, as unverified ORCID iDs will not appear in the published article. *Only*  the individual author can complete the verification step; PLOS staff *cannot*  verify ORCID iDs on behalf of authors.

We look forward to receiving your revised manuscript.

Kind regards,

Rodrigo Ramalho

Academic Editor

PLOS Global Public Health

Journal Requirements:

If the reviewer comments include a recommendation to cite specific previously published works, please review and evaluate these publications to determine whether they are relevant and should be cited. There is no requirement to cite these works unless the editor has indicated otherwise.

Please review your reference list to ensure that it is complete and correct. If you have cited papers that have been retracted, please include the rationale for doing so in the manuscript text, or remove these references and replace them with relevant current references. Any changes to the reference list should be mentioned in the rebuttal letter that accompanies your revised manuscript. If you need to cite a retracted article, indicate the article’s retracted status in the References list and also include a citation and full reference for the retraction notice.

# Author comment: Author response to Decision Letter 2

**DOI:** 10.1371/journal.pgph.0006551.r004

5 Aug 2026

Attachment Submitted filename: Response to reviewers comments 2.docx

# Editor report: Decision Letter 2

**DOI:** 10.1371/journal.pgph.0006551.r005

**Authors:** Rodrigo Ramalho

10 Aug 2026

Poverty Alleviation for People with Severe Mental Health Conditions in South Africa: A Qualitative Study

PGPH-D-26-01763R2

Dear Dr Asher,

We are pleased to inform you that your manuscript 'Poverty Alleviation for People with Severe Mental Health Conditions in South Africa: A Qualitative Study' has been provisionally accepted for publication in PLOS Global Public Health.

Before your manuscript can be formally accepted you will need to complete some formatting changes, which you will receive in a follow up email. A member of our team will be in touch with a set of requests.

Please note that your manuscript will not be scheduled for publication until you have made the required changes, so a swift response is appreciated.

IMPORTANT: The editorial review process is now complete. PLOS will only permit corrections to spelling, formatting or significant scientific errors from this point onwards. Requests for major changes, or any which affect the scientific understanding of your work, will cause delays to the publication date of your manuscript.

If your institution or institutions have a press office, please notify them about your upcoming paper to help maximize its impact. If they'll be preparing press materials, please inform our press team as soon as possible -- no later than 48 hours after receiving the formal acceptance. Your manuscript will remain under strict press embargo until 2 pm Eastern Time on the date of publication. For more information, please contact globalpubhealth@plos.org.

Thank you again for supporting Open Access publishing; we are looking forward to publishing your work in PLOS Global Public Health.

Best regards,

Rodrigo Ramalho

Academic Editor

PLOS Global Public Health

***********************************************************
